# Identification of a Novel Gene for Biosynthesis of a Bacteroid-Specific Electron Carrier Menaquinone

**DOI:** 10.1371/journal.pone.0028995

**Published:** 2011-12-14

**Authors:** Fuli Xie, Guojun Cheng, Hui Xu, Zhi Wang, Lei Lei, Youguo Li

**Affiliations:** State Key Laboratory of Agricultural Microbiology, Huazhong Agricultural University, Wuhan, People's Republic of China; University of New South Wales, Australia

## Abstract

Ubiquinone (UQ) has been considered as an electron mediator in electron transfer that generates ATP in *Rhizobium* under both free-living and symbiosis conditions. When mutated, the *dmtH* gene has a symbiotic phenotype of forming ineffective nodules on *Astragalus sinicus*. The gene was isolated from a *Mesorhizobium huakuii* 7653R transposon-inserted mutant library. The DNA sequence and conserved protein domain analyses revealed that *dmtH* encodes demethylmenaquinone (DMK) methyltransferase, which catalyzes the terminal step of menaquinone (MK) biosynthesis. Comparative analysis indicated that *dmtH* homologs were present in only a few *Rhizobia*. Real-time quantitative PCR showed *dmtH* is a bacteroid-specific gene. The highest expression was seen at 25 days after inoculation of strain 7653R. Gene disruption and complementation tests demonstrated that the *dmtH* gene was essential for bacteroid development and symbiotic nitrogen fixation ability. MK and UQ were extracted from the wild type strain 7653R and mutant strain HK116. MK-7 was accumulated under microaerobic condition and UQ-10 was accumulated under aerobic condition in *M. huakuii* 7653R. The predicted function of DmtH protein was confirmed by the measurement of methyltransferase activity in vitro. These results revealed that MK-7 was used as an electron carrier instead of UQ in *M. huakuii* 7653R bacteroids.

## Introduction

In bioenergetic chains, diverse chemical types of quinones, such as ubi-, plasto-, mena-, rhodo-, caldariella- or sulfolobus-quinones have been identified as membrane-bound, mobile hydrogen carriers in different species. Ubiquinone (UQ) is the dominant quinone species in α/β/γ-proteobacteria. The majority of strictly aerobic Gram-negative bacteria contain exclusively UQ [1], [2], [3]. Menaquinone (MK) is most widely distributed in most Gram-positive bacteria and anaerobic Gram-negative bacteria. Because MK is the only quinone in the early branching of phylogenetic tree archaeal and bacterial phyla, MK was speculated to be the ancestral type of quinone in bioenergetic systems [4]. It was deduced that the evolutionary transition of bioenergetic chains from high-potential MK to low-potential UQ in the proteobacterial phylum had drived by rising levels of dioxygen 2.5 billion years ago, and bioenergetic ambivalence of the respective organisms which work both on MK-and on UQ-pools, was necessary in transition proceed [5]. For example, *E. coli* and closely related species can switch between MK-and UQ-based bioenergetic chains in response to varying growth conditions. In *E. coli*, UQ and MK perform distinct functions. UQ-8 is used as the main quinone in aerobic respiration [6]. However, under anaerobic conditions, MK-8 and demethylmenaquinone-8 (DMK-8) are synthesized as electron transferring quinones [7], [8].

**Figure 1:**
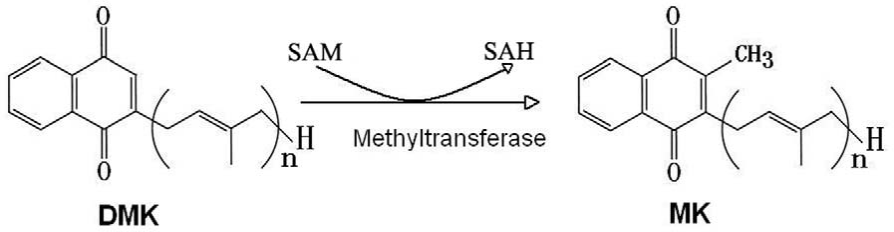
Methylation reactions in the biosynthetic pathway of MK. The length of the isoprenoid side chain (*n*) varies depending on the species. SAM, S-adenosyl-L-methionine; SAH, S-adenosyl-L-homocysteine; DMK, demethylmenaquinone; MK, menaquinone.

In most bacteria, MK is derived from chorismate which is comed from the shikimate pathway. Chorismate is initially converted into isochrismate and then into 2-succinyl-6-hydroxyl-2, 4-cyclohexadiene-1-carboxylate (SHCHC). SHCHC is dehydrated into o-uccinylbenzoate, and linked with CoA, then converted into 1,4-dihydroxy-2-naphthoate(DHNA). DHNA is added prenyl side chain into DMK, and DMK is converted into MK by S-adenosylmethionine-dependent methylation[9], [10]. In this pathway, DMK methyltransferase are encoded by *menG* genes (Figure1). Recently, an alternative microbial MK biosynthetic pathway was discovered whose genes have no homology to *men* genes, which were identified in *Streptomyces* [10]. In *E. coli*, the *ubiE* gene catalyzes the common C methylation step in MK and UQ biosynthesis [11]. The *E. coli* mutant strain AN70 containing the *ubi*E401 mutation lacks both UQ and MK and instead produces only DMK [12].

*Rhizobium*, classfied as the Gram-negative α-proteobacteria, is strictly aerobic under free-living but is microaerobic as bacteroids in nodules. *Rhizobium* contains diverse and complicated respiratory electron transport systems to cope with free-living and symbiosis conditions. Under aerobic conditions, *BradyRhizobium japonicum* transfers electrons from the ubiquinone (UQ) pool to the O_2_-reducing *aa*_3_-type terminal oxidase via the FeS protein-cytochrome *bc*_1_ complex and a membrane-bound cytochrome *c* [13], [14]. Under microaerobic and bacteroid conditions diverse electron transport pathways were reporteds for *B. japonicum*, and one of the mainstream chains was that a *cbb*_3_-type-terminal oxidase transferred electrons from Fes/*bc*_1_ to O_2_ [15]. In a different study, when nitrous oxide was supplied as the terminal electron acceptor, electrons flowed from the UQ pool to nitrous oxide via cytochrome *bc*_1_ [16]. In the electron transport pathway of *Rhizobium trifolii*, flavoprotein lies between NADH and UQ, and *b*-cytochromes transfer electrons from UQ to O_2_ [17]. These reports suggested that UQ was an electron mediator to oxidize NAD(P)H in *Rhizobium* cultured under different conditions.

*Mesorhizobium huakuii* can establish a nitrogen-fixing symbiosis with *Astragalus sinicus* and form indeterminate nodules. *M. huakuii* has a strict specificity for *A. sinicus* which makes this species important for studies on the molecular mechanism of symbiotic nitrogen fixation. We previously constructed a transposon mutagenesis library by random insertion of the Tn5-B12S into the *M. huakuii* 7653R genome [18]. A new gene, *dmtH*, was isolated from the mutagenesis library which mutant formed ineffective nodules on *A. sinicus*. The DNA sequence and conserved protein domain analyses revealed that *dmtH* encodes DMK methyltransferase, which catalyzes the terminal step of MK biosynthesis. The result is interesting,because no *Rhizobium* has been reported to operate electron transfer containing both MK and UQ thus far. We hypothesized that a few of *Rhizobia* may use MK as electron carrier under symbiosis conditions. In this study, we identified *dmtH* function by symbiotic phenotype and protein enzymatic activity assays in vitro. The results suggested that *dmtH* encoded a DMK methyltransferases and MK was biosynthesized in *M. huakuii* bacteroids. The implications of these findings for the functions of *dmtH* and MK in bacteroid metabolism and symbiosis are also discussed.

## Results

### Cloning and sequence analysis of the dmtH gene

The flanking sequence of the transposon insertion site was amplified by TAIL-PCR, from the mutant strain HK115 (nod^+^fix^−^). After sequencing, a homology search revealed that a consensus sequence was present only in some isolates of *Rhizobium*. Since the genome of *M. huakuii* has not still been sequenced, a pair of specific primers, *dmt*UP and *dmt*LOW were designed, which were based on the homologous sequences of *M. loti* MAFF303099, to amplify a 750-bp fragment including the complete ORF sequences from the genome of wild-type strain 7653R. This gene was designated as *dmtH* (GenBank accession no. JN400272). The *dmtH* gene is predicted to encode a 249-amino acid polypeptide with an expected molecular mass of 27.3 kDa and a pI value of 5.4. The homology analysis of the DmtH protein showed that had 99% identity to the function-unknown gene *mll2332* from *M. loti* MAFF303099, and another similar proteins were annotated as demethylmenaquinone methyltransferase proteins in *M. opportunistum* WSM2075 (94% identity), *R. leguminosarum* bv. *trifolii* WSM1325 (90% identity) and *R. etli* CFN42 (88% identity). DmtH homology protein also presented in other bacteria as annotated demethylmenaquinone methyltransferase, such as *Amycolatopsis mediterranei* U32 (59% identity), Roseiflexus sp. RS-1 (49% identity) (Figure S1, S2). The *dmtH* gene was also discovered that had no homology to *men* genes, whereas had 47% identity and 64% Positives with annotated demethylmenaquinone methyltransferase gene of *Streptomyces lividans* TK24. In *Streptomyces*, MK biosynthetic pathway was identified and whose genes have no homology to *men* genes(10). We thus assumed that the DmtH protein encoded DMK methyltransferase, which catalyzes methylation at the terminal step in MK biosynthesis.

### Construction of dmtH null mutant HK116 and symbiotic phenoytpe identification

**Figure 2:**
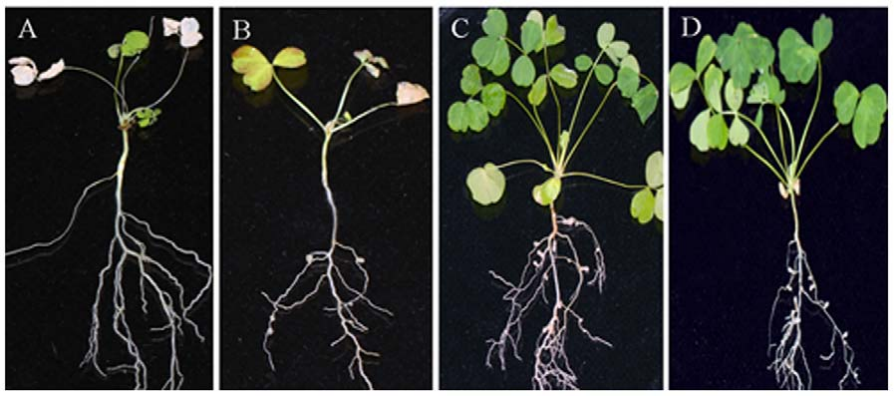
Plant growth test of the symbiotic ability of the disruptant HK116 on *A. sinicus*. (A) Control plant not inoculated *Rhizobium*. (B) Plant inoculated by HK116 formed pseudonodules and yellow leaves. (C) Control plant inoculated by the wild type 7653R. (D) Plant inoculated by complemented strain HK116C restored nitrogen-fixing ability.

To confirm that the function of the *dmtH* gene in nodulation and nitrogen fixation ability was disrupted by the transposon insertion, a *dmtH* gene null mutant was constructed by the homologous recombination method, and one mutant strain, HK116, which formed ineffective nodules on *A. sinicus*, was obtained. The disruption of *dmtH* in mutant strain HK116 was verified by PCR. The symbiotic phenotype of the disruptant HK116 was examined by plant growth test (Figure 2 and Table 1). The results showed that HK116 formed small abnormal white nodules with a host plant, but lacked nitrogen-fixing ability. Nitrogenase activity of the nodules induced by HK116 was undetectable, and the number of nodules remarkably decreased compared to wild type 7653R.

**Table 1:** Symbiotic phenotypes of *M. huakuii* wild type strain 7653R, mutant strain HK116 (Nod^+^ fix^−^), and complemented strain HK116C.

Strain M. huakuii	Plant dry wt. (mg plant^−1^)	No. of nodules per plant	Nodule fresh wt. (mg plant^−1^)	Acetylene reduction activity(nmol of ethylene plant^−1^ h^−1^)
7653R	44.28^a^	21.50^a^	27.38^a^	2.55^a^
HK116	12.45^b^	11.08^b^	6.9^b^	0
HK116C	43.71^a^	22.44^a^	26.81^a^	2.47^a^
Control	11.08^b^	0	0	0

*Data are the average of five replicates

a,b Values in each column followed by the same letter are not significantly different (P<0.05).

Control: plants not inoculated with rhizobia strain.

### Microscopic analysis in nodules

**Figure 3:**
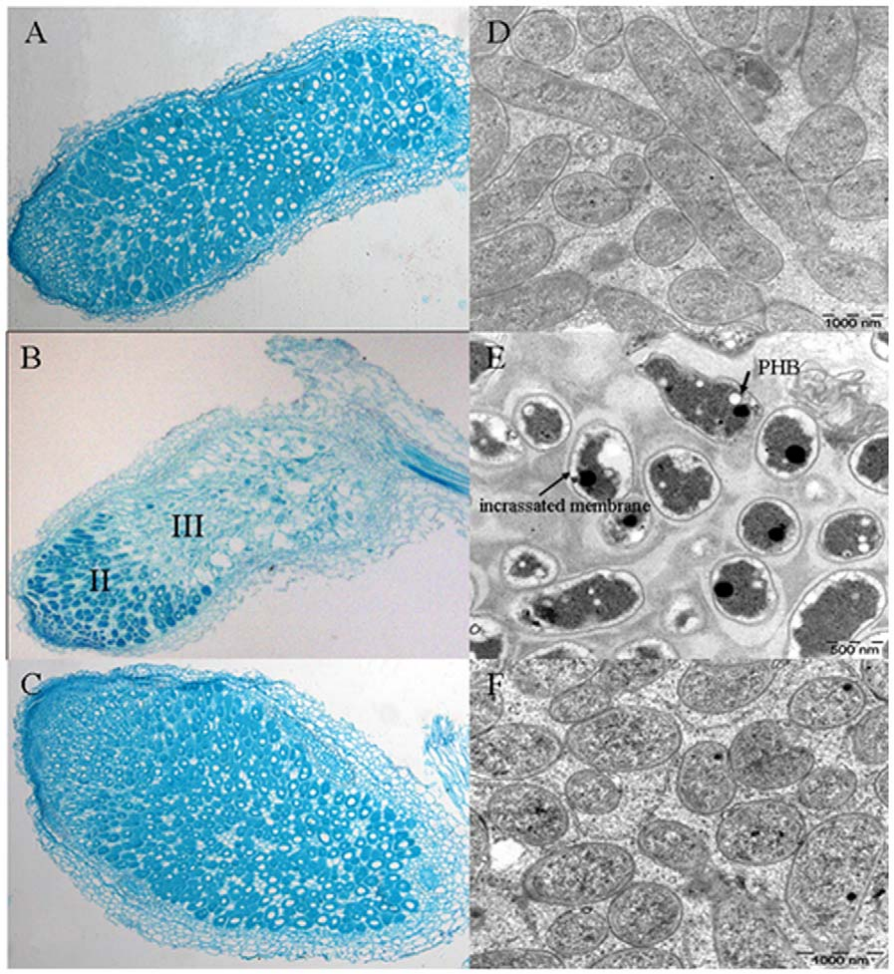
Paraffin section and ultrastructure observations of 5-week-old nodules formed by wild type strain 7653R, disruptant HK116, and complemented strain HK116C. (A) Paraffin section of normal nitrogen-fixation nodules formed by wild type strain 7653R. (B) Paraffin section of nodules induced by disruptant HK116 strains in which the *dmtH* gene was disrupted and nitrogen-fixation ability lost; bacteroids are mainly located in II zone. (C) Paraffin section of nodules induced by the complemented strain HK116C with restored nitrogen-fixation ability. (D) Ultrastructure of normal bacteroids in nodules induced by wild-type 7653R. (E) Ultrastructure of aberrant bacteroids in nodules induced by disruptant HK116, with smaller size, incrassated membrane, and visible PHB granules in bacteroid cells. (F) Ultrastructure of function-restored bacteroids in nodules induced by the complemented strain HK116C.

Paraffin section of the nodules induced by HK116 showed a clearly decreased number of bacteroids in nodules (Figure 3). Since a few symbiosomes were contained in the nodule infection zone (II zone) and the II–III interzone, further ultrastructural comparisons between nodules induced by HK116 and by 7653R were performed by transmission electron microscopy. Consistent with the light microscope observation, bacteroids were mainly located in the II zone and were degraded in the nitrogen fixation zone (III). Many symbiosomes in the nodules infected by HK116 were aberrant and the bacteroid membrane showed incrassation. Bacteroid sizes were smaller than in the wild type nodules. In the bacteroids, much poly-β-hydroxybutyrate (PHB) was distinctly observed (Figure 3). These changes showed that the *dmtH* gene is necessary to achieve efficient symbiotic interaction between *M. huakuii* and *A. sinicus*.

### Complementation of the disruptant HK116

Complementation studies were carried out by cloning the *dmtH* gene into the broad host range vector pBBRgus. The disruptant strain HK116 transformed with pBBRgus-*dmtH* was inoculated on *A. sinicus*. HK116 harboring pTRgus-*dmtH*, which was designated HK116C, formed normal nodules and restored nitrogen-fixing ability to the host plant (Table 1). Light and transmission electron microscopy showed that bacteroids in root nodules induced by HK116C had similar numbers and configuration to wild type nodules, except for a slightly smaller size (Figure 3).

### Quantification of dmtH gene expression by real-time RT-PCR

**Figure 4:**
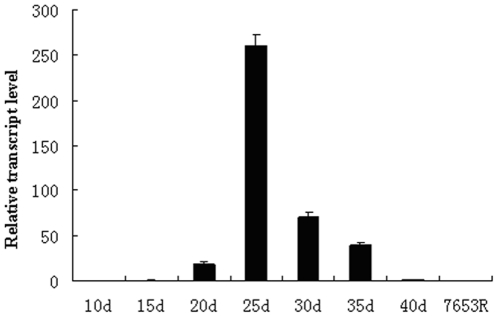
Expression patterns of *dmtH* gene in free-living cells and symbiotic nodules. Gene expression levels were examined by real-time RT-PCR. Nodules were collected on different days after inoculation with 7653R. Expression levels of *dmtH* were highest in nodules at 25 dai, and hardly expressed in free-living cells. Histograms represent quantification of the products normalized to the constitutive control *rnb*. The experiment was repeated three times.

The relative expression level of the *dmtH* gene in root nodules collected at several time points after inoculation, and in free-living cultures was estimated by quantitative real-time PCR (Figure 4). The results show that the *dmtH* gene had much lower expression levels under free-living conditions, and was specifically expressed in bacteroids during symbiosis. The expression of the *dmtH* gene achieved the highest level in nodules at 25 days after inoculation (dai). These results indicated that *dmtH* gene expression was induced by symbiosis, and was indispensable for bacteroid development and nitrogen fixation, but was not needed for the free-living growth and metabolism of *Rhizobia*.

### Analysis of menaquinone and ubiquinone in 7653R and mutant HK116

**Figure 5:**
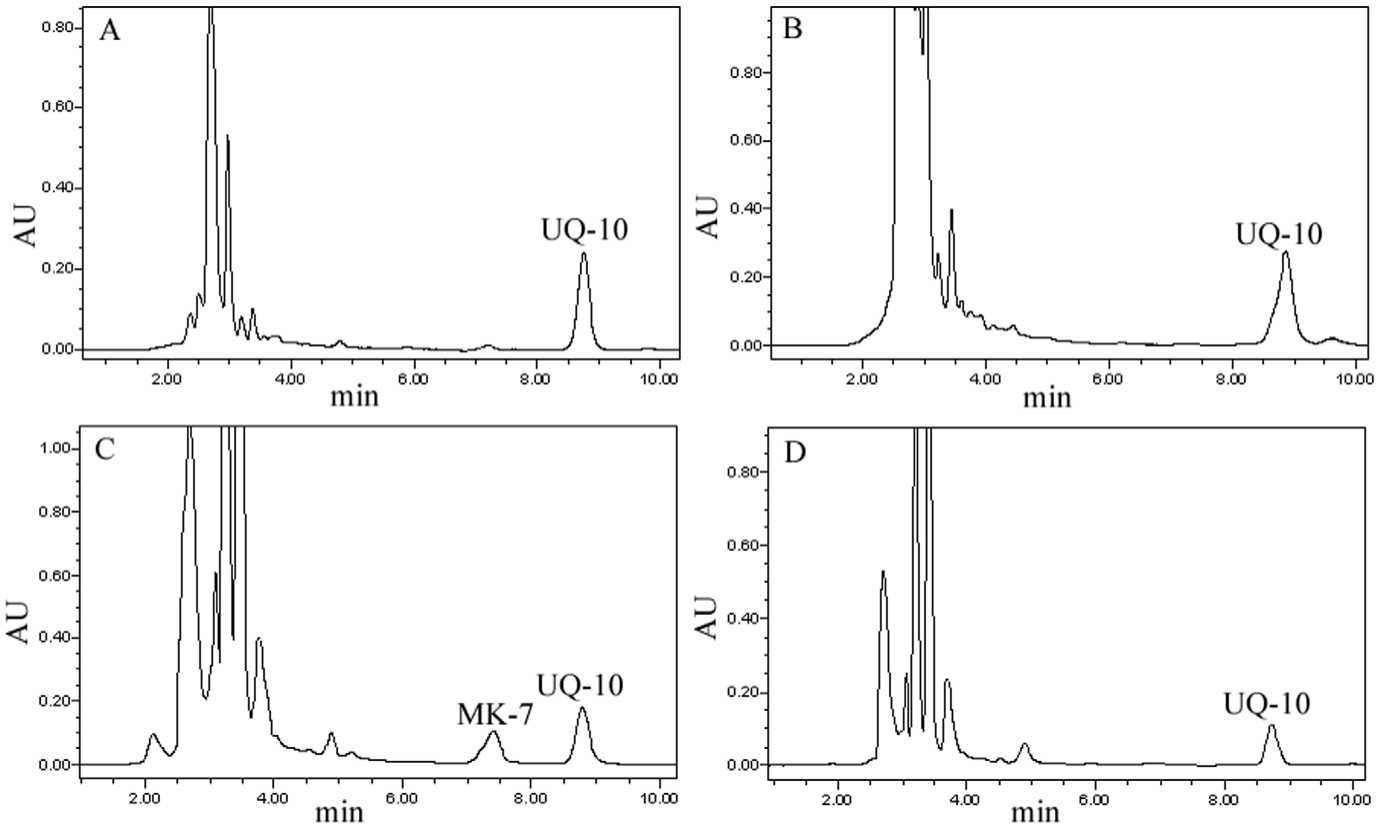
HPLC analyses of MK and UQ from wild type strain 7653R and disruptant HK116 under aerobic and microaerobic conditions. (A) UQ-10 was accumulated in 7653R a under aerobic condition. (B) UQ-10 was accumulated in HK116 under aerobic condition. (C) MK-7 was accumulated in wild type strain 7653R when culturing conditions were shifted to microaerobic for 24 hours. (D) MK-7 was not produced in disruptant HK116 under microaerobic condition.

Recent studies have shown no evidence of the presence of MK in *Rhizobium*. The function of DmtH protein was hypothesized to participate in the biosynthesis of MK in 7653R, and the *dmtH* gene was specifically expressed in bacteroids. Therefore, we compared and identified the electron carrier used under free-living and symbiotic conditions. Quinone was extracted from 7653R and mutant HK116 growing under aerobic and microaerobic conditions and extracts were analyzed by reverse-phase high-performance liquid chromatography (HPLC) and mass spectrometry (MS). The results demonstrated that UQ-10 accumulated in 7653R and HK116 under aerobic conditions, but when growing conditions were switched from aerobic to microaerobic, MK-7 was confirmed to be produced in the wild type strain 7653R but was not in the extract from mutant HK116, in which the content of UQ-10 was also significantly decreased (Figure 5), and the result of MS further confirmed MK-7 and UQ-10 were accumulated in 7653R in microaerobic respiration. These results indicated that MK was synthesized only in bacteroids of wild type 7653R and not produced in free-living cells, implying that the bacteroids use MK as the specific electron carrier under symbiosis conditions to accommodate metabolic changes during development and nitrogen fixation.

### Methyltransferase activity measurement of DmtH protein in vitro

**Figure 6:**
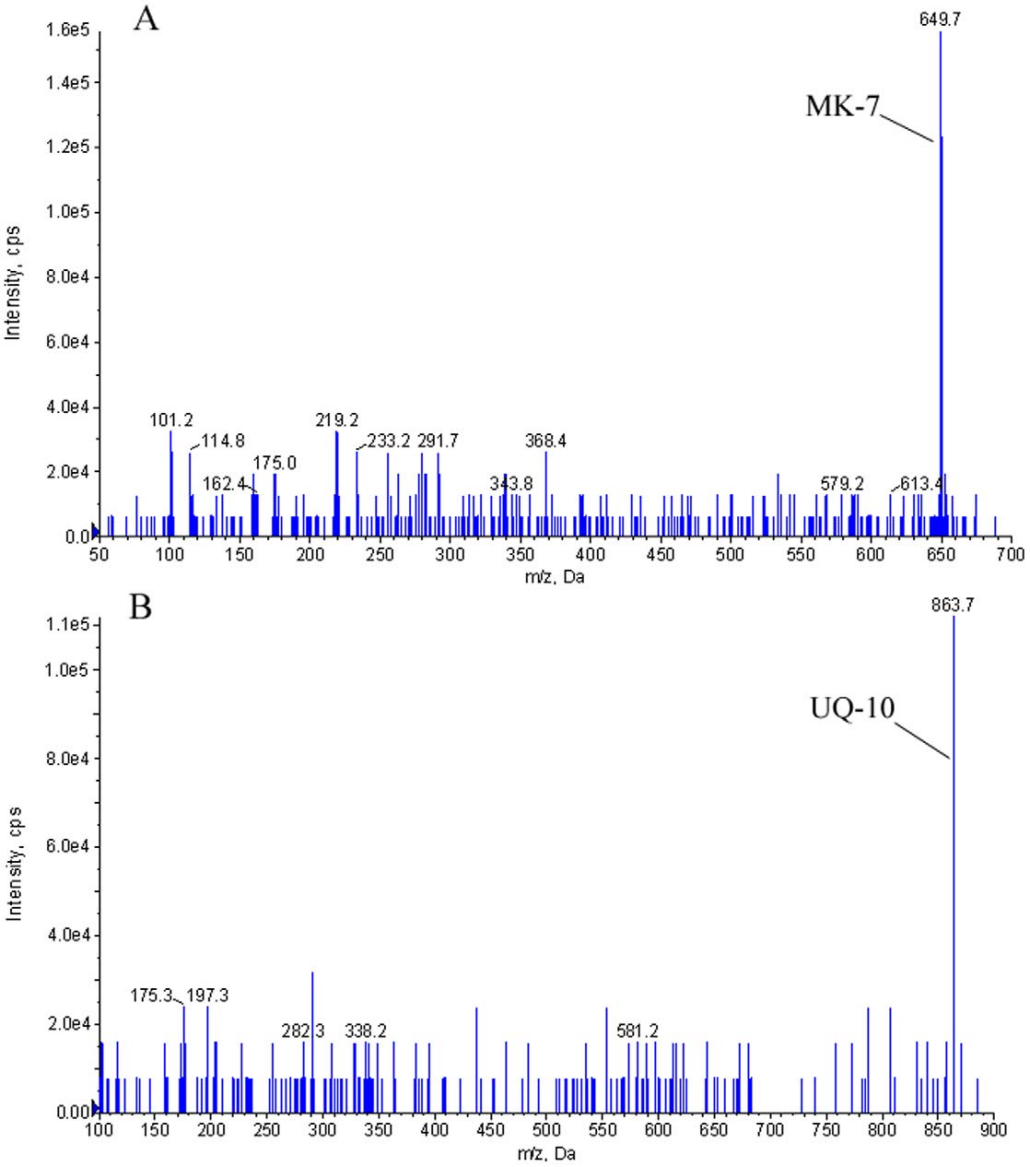
Mass spectrum of quinine compounds from *M. huakuii* 7653R cultured under microaerobic condition. (A) The mass spectrum of MK7 which showed that MK7 was accumulated in *M. huakuii* 7653R under microaerobic respiration. (B) The mass spectrum of UQ-10 which showed that UQ-10 was accumulated in *M. huakuii* 7653R.

To identify the enzymatic activity of the DmtH protein as a DMK methyltransferase that catalyzes methylation in MK biosynthesis, an overexpression plasmid pET*dmt* for the *dmtH* gene was constructed and transformed into recipient strain *E. coli* Rosetta 2(DE3). The target protein was overexpressed after inducing with isopropyl β-D-thiogalactopyranoside and purified (Figure 6).

**Figure 7:**
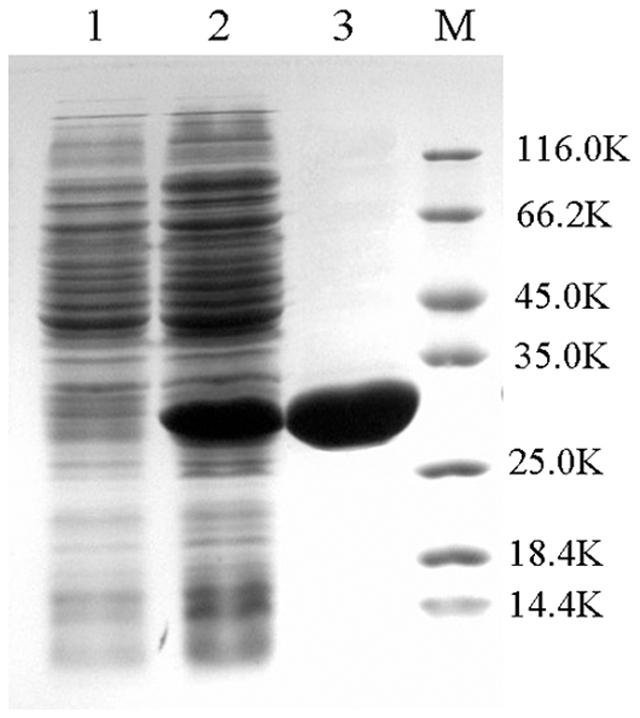
Overexpression of the *dmtH* gene in *E. coli* and target protein DmtH purification. The DmtH protein was separated on an SDS-polyacrylamide gel. *E. coli* Rosetta 2(DE3) harboring pET28a was used as the control (lane 1). *E. coli* Rosetta 2(DE3) harboring pET*dmt* was incubated with 28 KDa DmtH protein (lane 2). DmtH protein was incubated after purification (lane 3). Protein markers are on the right.

**Figure 8:**
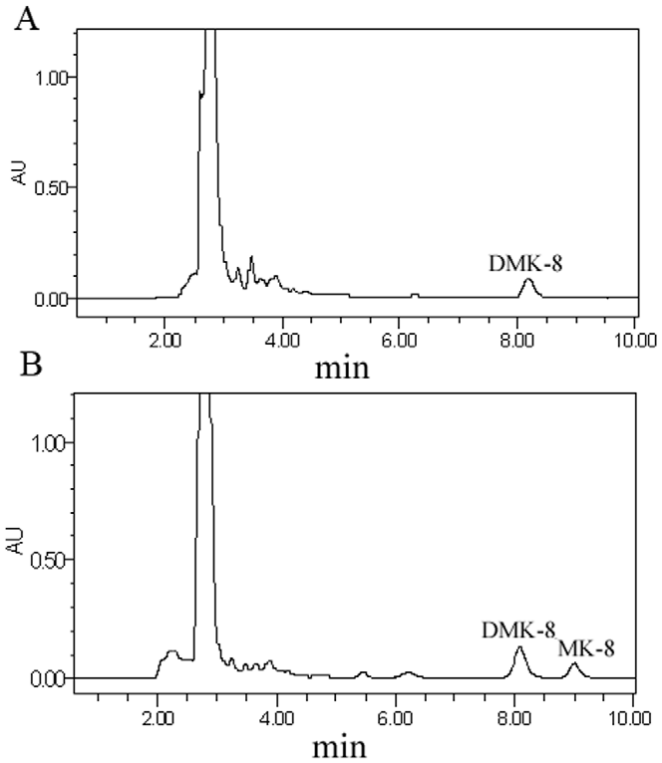
HPLC analyses of quinone compounds for testing DmtH peotein activity. (A) DMK-8 was extracted from *E.coli* JC7623**Δ**4-1(*ubiE*: *Kan* ^r^) but MK-8 was not formed; (B) MK-8 was synthesized in vitro in the reaction system by purified DmtH protein, SAM and *E.coli* JC7623**Δ**4-1 cell homogenate at 37°C for 1 h.

Because the substrate specificity of the methyltransferase is not stringent for the prenyl side chain of demethyl-MK [9], [19], DMK-8 of *E.coli* JC7623**Δ**4-1, which accumulated only DMK-8 but not MK-8 (Figure 7A), was used as the substrate of DmtH protein activity test. A crude homogenate of *E.coli* JC7623**Δ**4-1 was prepared. S-adenosyl methionine (SAM) was used as the methyl donor. The reaction system, with the crude *E.coli* JC7623**Δ**4-1 homogenate, purified DmtH protein and SAM, was incubated at 37°C for 1 h, and the products analyzed by HPLC and MS. MK-8 was found in the reaction products in addition to DMK-8 (Figure 7B, 8 and Figure S5). This showed that DmtH had methyltransferase activity to transfer a methyl group from SAM to DMK-8, indicating that the *dmtH* gene had a function similar to the *ubiE* gene.

## Discussion

In 1960 s–1980 s, studies were carried out on the electron transfer mechanisms of *Rhizobium*. UQ was considered to be the electron carrier operating electron transfer in free-living cells and bacteroides. In vitro, menadione could substitute for UQ to reduce cytochrome *b* [20], but no evidence was obtained for the presence of MK in *Rhizobia* [21]. In previous reports, work focused on the electron transfer pathway of *BradyRhizobium* sp., but with little on *Mesorhizobium*.

In this work, the function of the *dmtH* gene was identified and characterized by gene disruption, symbiotic phenotype, functional complementation, gene expression pattern and DMK methyltransferase activity measurement in vitro. The results showed that UQ-10 accumulated under aerobic conditions, while synthesis of MK-7 was induced under microaerobic conditions in strain 7653R when NO_3_^+^ was supplied as terminal electron acceptor. Since the *dmtH* gene was mainly expressed during symbiosis, we assume that MK-7 was produced only in the bacteroids with little in free-living cells.

Also supporting MK as a quinone pool in bacteroids was the ultrastructural of nodules induced by HK116, in which PHB was accumulated and the membrane in bacteroids was also incrassated. *M. huakuii* form indeterminate nodules on host plants and do not accumulate visible PHB granules during symbiosis. PHB granules are synthesized during the initial stage of invasion and used in the process of bacteroid differentiation [22]. Intracellular PHB may fuel cell division and growth during root infection and invasion [23]. PHB granules accumulating in bacteroids implied that the metabolism of the carbon source or energy was shifted. Normally, intracellular PHB as a carbon and energy source can be metabolized in the TCA cycle for NADH production, which then flows into the electron transfer chain for ATP formation during root infection and invasion. However, in the symbiosis of the disruptant HK116, MK could not be synthesized, which led to obstruction of the electron transfer chain. To allow continued operation of the TCA cycle, NAD(P)H was channeled into other biosynthesis reactions, such as PHB synthesis, for use as reducing equivalents.

During nitrogen fixation, the bacteroid is supplied with C_4_-dicarboxylates as a carbon resource by the plant [24], and bacteroids carry the carbon resources into the TCA cycle to produce reducing equivalents and ATP for driving nitrogen fixation [25], [26]. Therefore, NADH synthesized from metabolites supplied by host plant, did not flow into the electron delivery chain to produce ATP for nitrogen fixtion in the disruptant HK116 bacteroids lacking DMK methyltransferase. Instead, the NADH was used for synthesis of PHB and long-chain fatty acids, which further facilitated their accumulation. Thus, abnormal morphologies and structures of bacteroids, such as smaller size, membrane incrassation and PHB granules, were observed (Figure 4). Therefore, the obstruction of MK biosynthesis and the electron transfer chain in bacteroids, caused impairment of the generation of ATP, and *Rhizobia* could not carry out the normal differentiation program in nodules, losing nitrogen fixation ability.

In *E. coli*, UQ is the electron-transferring quinone in aerobic respiration, and MK in anoxic conditions. Correspondingly, two dehydrogenases that transfer electrons from NADH to the respiratory chain are present in cells. NDH-1 type dehydrogenase, which is a multisubunit complex, is expressed under anaerobic conditions, reducing fumarate and using MK as an electron acceptor. NDH-2 dehydrogenase, a monomeric protein smaller than NDH-1, oxidizes succinate and reduces UQ [27], [28], [29]. If MK was used as an electron carrier in bacteroids of *Rhizobia*, two dehydrogenases would also be expected to participate in oxido-reduction reactions in free-living and symbiosis modes, as reported for *R. leguminosarum* [30]. This study isolated two different NADH-dependent dehydrogenses, DH1 and DH2, from *R. leguminosarum*. The DH1 complex, like the NDH-1 type dehydrogenases with a molecular mass of 550 kDa, is a specific-bacteroid membrane component. The DH2 complex, with a molecular mass of 110 kDa, is present in both bacteroids and free-living bacteria. This implies that MK could act as an electron carrier in bacteroids. In agreement with our findings, the *dmtH* gene was distributed only in a few of *Rhizobium* genomes including *Mesorhizobium* sp., *R. leguminosarum* and *R. etli*, by homology analysis and Southern blotting (Figure S3), which showed that MK was not a commonly trait in most *Rhizobia*.

In previous report, MK was considered to represent the ancestral type of quinone in proteobacteria, and MK-to-UQ transition which had occurred 2 billion years ago probably proceeded via ambivalent organisms which can use both types of bioenergetic chains[5]. The phenomena was observed in *E. coli* and closely related species. Recently the γ-proteobacterium *Shewanella* ANA-3 was reported to perform bioenergetic electron transport from lactate to arsenate based on a MK-pool[31]. The *Halorhodospira* species *H. halophila*, one of photosynthetic members, was also proved to contain both UQ and MK, and operate the photosynthetic cycle based on MK-pool which was not a common trait of *Halorhodospira/Ectothiorhodospira*, and other subclasses of *Halorhodospira* worked on UQ bioenergetic chains when grown phototrophically [5]. According to the findings, we guessed *M. huakuii* maybe one of ambivalent organisms. In proceeding of the *Rhizobium* evolvement, the difference of host plants appears to influence bacteroid physiological transition. *Rhizobium* could form indeterminate nodules in galegoid legumes and deteterminate nodules in nongaleoid legumes. Using recombinant *Rhizobium* strains nodulating both two legume types, bacteroid cell cycle and differentiation had shown to be controlled by the host plant in the *Rhizobium*–legume symbiosis [32]. *M. huakuii* has a strict host specificity and only form nodules in *A. sinicus* which imply *M. huakuii* taking on lower evolution status in all *Rhizobia*,therefore still perform energy-conserving chains based on MK pools.

*Rhizobium* have complicated branched electron transfer networks that enable them to grow under different environments, such as the four branched respiratory chains found in *B. japonicum* [15], and two branched pathways in *Rhizobium trifolii* [17]. In our studies, MK-7 was confirmed to play an important role as a quinone pool in *M. huakuii* bacteroids, which implies that more complicated branched electron transfer networks exist in *Rhizobia*. Detailed information on the full respiratory chain of *Mesorhizobia*, particularly the pathway used under symbiosis, requires further research.

## Materials and Methods

### Media and growth conditions

*E. coli* strains were cultured in Luria-Bertani (LB) medium at 37°C. *Rhizobia* were grown in tryptone yeast medium (TY) at 28°C. When required, the following antibiotics were used: ampicillin (Ap) 100 µg/ml; spectinomycin (Spe) 50 µg/ml; tetracycline (Tet) 15 µg/ml; gentamicin (Gm) 20 µg/ml; and kanamycin (Kan) 50 µg/ml.

### Cloning and sequence analysis of dmtH gene

The genome of *M. huakuii* has not still been sequenced at present, hence thermal asymmetric interlaced PCR (TAIL-PCR) was used to amplify the flanking regions of Tn5-B12S from the genome of the original symbiosis defective mutant HK115 (nod^+^fix^−^). Techniques were performed as previously described [18]. TAIL-PCR products were cloned into plasmid vector pMD-18T (Tarkara, DaLian, China) and sequenced. This gene was designated as *dmtH* (GenBank accession no. JN400272). Two oligonucleotides, *dmt*UP (5′-ATGACGCATACCTCCGACATC-3′) and *dmt*LOW (5′-CTAATTTGGGTTCCCCAAGC-3′) were designed to amplify the 750-bp DNA sequences containing the full-length ORF of *dmtH* gene from total DNA of *M. huakuii* 7653R. Nucleotides and protein homology searches were carried out by BLAST analysis (http://www. ncbi. nlm. nih.gov/BLAST). The protein-conserved domains were predicted using the InterProScan server of EBI (http://www.ebi.ac.uk/InterProScan).

### Construction of dmtH null mutant

Primers used to PCR-amplify a segment of 7653R genomic DNA containing the *dmtH* ORF with 200 bp of flanking sequence on either side of the coding sequence were: *dmt*UP1: 5′-TCAATTTCAACCGGAGTCC-3′; *dmt*LOW1: 5′-GACCTGGTTTCGCGCGCG-3′. The resulting PCR product (1.1 kb) was ligated into pMD-18T to generate pMD*dmtH*. A 950-bp fragment containing a Km-resistance cassette was amplified from plasmid pMH1701 by PCR with primers kanNcoIU (5′-TTTCCATGGGCAAAGCCACGTTGTGTCT-3′; the NcoI restriction site is underlined ) and kanNcoIL (5′-TTTCCATGGAGAAAAACTCATCGAGCA-3′; the *Nco*I restriction site is underlined). The Km-resistance cassette was inserted into the unique *Nco*I site of the *dmtH* gene in pMD*dmtH* to create plasmid pMD*dmtH*-Km. A 2.1-kb fragment containing *dmtH* and the Km cassette was cut from pMD*dmtH*-Km and subcloned into pJQ200 mp18 using *Pst*I and *Xba*I restriction sites. The resulting suicide plasmid, pJQ*dmtH*-Km, was conjugated into *M. huakuii* 7653R by tri-parental mating with a helper plasmid pRK2013 [33]. A *dmtH* double-crossover recombinant was selected using the sac mutagenesis strategy as previously described [34]. The disruption of *dmtH* in mutant strain HK116 was verified by PCR and plant nodulation experiment.

### Complementation

For the symbiotic function complementation test, the *dmtH* gene was amplified with 7653R total DNA as the template using the following two primers: *dmt*UP2: 5′- ACTCTCGAGTAATCTAGACTGAACGAGGAGGTCGACGATGACGCATAC-3′ (the *Xho*I restriction site is underlined), *dmt*LOW2: 5′- ACTCTCGAGCTAATTTGGGTTCCCCAA -3′ (the *Xho*I restriction site is underlined). The amplified fragment digested with *Xho*I was cloned into the previously modified vector pBBR*gus*, in which *gusA* was inserted into the broad-host-range plasmid pBBR1MCS5 under the Lac promoter [35]. The recombinant plasmid pBBR*gus*-*dmt* was obtained, and transferred into mutant strain HK116 by triparental mating. Transconjugants were used to inoculate seedlings of *A. sinicus* to test for ability to restore the symbiotic phenotype of HK116.

### Plant growth and nodulation experiment

Seeds of *A. sinicus* cultivar XY202 were surface sterilized for 5 min in 75% ethanol, soaked for 20 min in 2% sodium hypochlorite, and rinsed extensively with sterile water. Plants were grown in pots filled with sterile sand containing nitrogen-free Fahraeus solution [36]. Cultivation was carried out in a controlled-environment chamber (18 h light and 6 h darkness at 22 and 20°C, respectively) for 35 days. Nodules were collected for paraffin-embedded section slides, electron microscopy and nitrogenase activity examination. Nitrogenase activity was measured by gas chromatography (GC) as acetylene reduction activity as previously described [37].

### Microscopic analysis

Light and transmission electron microscopic studies of nodules were performed as follows. For paraffin sections, root nodules were dehydrated in ethanol and xylene, embedded in paraffin wax, serial-cut longitudinally, and slides were stained with toluidine blue, then observed with a light microscope. For electron microscopy, after nodules were fixed with 2.5% glutaraldehyde, postfixed in 1% osmium tetroxide, rinsed, dehydrated and embedded in London resin white, ultra-thin sections were taken and examined in a Hitachi H-7650 transmission electron microscope.

### Quantification of dmtH gene expression by real-Time RT-PCR

To determine the relative *dmtH* gene expression level, total RNA was isolated with Trizol reagent from free-living *M. huakuii* 7653R cultured in TY liquid medium and plant nodules, which were harvested from *A. sinicus* inoculated with wild type strain 7635R after 10, 15, 20, 25, 30, 35 and 40 days. Quantitative real-time PCR was performed using the SYBR Premix ExTaq (Takare, Dalian, China) on the iCycler iQ5 Multicolor Real-Time PCR Detection System (primers: 5′-CATCTATCCCAATGCCGTCAAC-3′ and 5′-CGAGAACTCTTCCCAATCGTG-3′) (Bio-Rad, Hercules, CA, USA). Expression levels were normalized using rnpB gene as internal control (primers: 5′- AAGGCCGCAAGTGAGGAAAGTC-3′ and 5′-GGTTTACCGTGCCGCTCCTGTTG-3′). Data were analyzed by the relative quantification method (2^−ΔΔCT^) to calculate expression and reactions were performed in triplicate.

### Extraction and analysis of MK and UQ in 7653R and mutant HK116

Cells grown for extraction were cultured aerobically in a 500-ml flask containing 100 ml of basal medium (glucose 20 g/l, peptone 5 g/l, yeast extract 3 g/l, malt extract 3 g/l ) at 28°C and 200 rpm for 20 h, then shifted to low O_2_ by sealing the flask with a stopper through which a cannula was inserted to allow continuous sparging of the culture with a compressed gas mixture of prepurified N_2_ (>99%), the indicated amount of O_2_, and 10 mM KNO_3_, which was added to the culture under microaerobic conditions. After culturing for 36 hours microaerobically, cells were harvested by centrifugation, and quinones were extracted with methanol/ethyl acetate (1∶1) and evaporated in vacuo [38]. Lipid extracts were resuspended in 1 ml of ethyl acetate per 3 liters of culture. The obtained materials were analyzed HPLC (Waters) and MS (API5000) with an isopropanol-methanol (1∶1) mobile phase and quinones were monitored by UV/Vis absorption spectroscopy at 248 and 270 nm. Standards of MK4, MK-7, MK9 and UQ-10 were used as controls (Figure S4) (Seebio, China).

### Methyltransferase activity assay of DmtH protein in vitro

To obtain adequate amounts of target protein for activity measurements, we constructed the overexpression plasmid pET*dmt* for the *dmtH* gene. Two oligonucleotide primers 5′-CTCGAGTCTAGAAATAATTTTGTTTAACTTTAAGAAGGAGATATACGATGACGCATACC-3′ (the *Xba*I restriction site is underlined) and 5′- ATAACTCTCGAGATTTGGGTTCCCCAA-3′ (the *Xho*I restriction site is underlined) were synthesized, and used to amplify *dmtH*. PCR products were cloned into the pET28a vector digested with *Xba*I and *Xho*I. The resulting plasmid, named pET*dmt*, was transformed into *E. coli* Rosetta 2(DE3) for the overexpression of the target protein DmtH. Expression, extraction and purification of DmtH protein was carried out by standard methods on Bio-Rad Biologic LP apparatus [39].

*E. coli* JC7623**Δ**4-1, which contains only DMK but no MK, was kindly supplied by Professor Catherine F. Clarke of University of California. Crude homogenates of *E. coli* JC7623**Δ**4-1 cells were prepared as a reaction substrate for DmtH protein activity assays. As described previously, JC7623**Δ**4-1 cells grown into late exponential phase in LB medium were centrifuged, suspended in a solution of 25 mM Tris-HCl buffer pH 7.7 containing 1 mM EDTA and 10 mM 2-mercaptoethanol (2 ml/g of wet cells), and disrupted with a Branson sonifier [9].

The DmtH protein reaction mixture contained, in a total volume of 10 ml, 4 ml of crude homogenate of JC7623**Δ**4-1 cells, 0.1 M Tris-HCl buffer (pH 8.0), 10 mM MgCl_2_, 10 mM dithiothreitol, 10% aqueous Triton X-100 (to give a final concentration of 0.5% Triton X-100), 10 nmol S-adenosyl-L-methionine (SAM) and 1 µM *dmt*H product. After incubation at 37°C for 1 h, the reaction was stopped by addition of 10 ml of 0.1 M acetic acid in methanol. The reaction products were extracted with 40 ml methanol/chloroform (1∶1) and evaporated in vacuo. The lipid extracts were resuspended in 0.5 ml of chloroform/liter of culture, and analyzed by HPLC and MS.

## Supporting Information

Figure S1 **Multiple sequence alignment of the DmtH protein and the homologous sequence.** Columns that are dark represent identical residues, lighter black represent similar residues in function. The numbers on the upper indicate the positions of amino acids. Protein names are indicated to the left of the alignment. These proteins come from *Moserhizobium. loti* MAFF303099 (mlr2332), *Moserhizobium opportunistum* WSM2075 (MesopDRAFT_4419), *Rhizobium leguminosarum* bv. *viciae* WSM1325 (RLeq4868), *Rhizobium etli* CFN42 (RHE_PE00095), *Amycolatopsis mediterranei* U32 (AMED3148), *Roseiflexus* sp. RS-1 (RoseRS_2046), *Thermomicrobium roseum* DSM 5159 (trd_1944), *Roseiflexus castenholzii* DSM 13941 (Rcas_3023), *Streptomyces lividans* TK24 (SlivT_010100003505), *Streptomyces ghanaensis* ATCC 14672 (SSFG_00363), *Burkholderia phytofirmans* PsJN (Bphyt_4892).
(TIF)

Figure S2 **Homology tree of DmtH homologs.** The homology tree was constructed using MEGA5.04 software (Tamura K, 2011). The scale bar indicates 10% substitutions per site.
(TIF)

Figure S3 **Southern blot analysis of** ***dmtH*** **gene in different** ***Rhizobium*** **genomes.** The total genomic DNA of tested strains were digested for at least 3 h with 5-10 U of restriction enzyme PstI and BamHI per µg of DNA. Digested genomes were subjected to electrophoresis and transfer to nylon membranes, then hybridized to a ^32^P-labeled probe. The tested strains was *Agrobacterium tumefaciense* IAMI 3129 (A. tum), *Sinorhizobium medicae* USDA 1037 (S. med), *Sinorhizobium meliloti* USDA 2011 (S. mel), *Sinorhizobium saheli* LMG 7837 (S. sah), *Rhizobium leguminosarum* LRP 5045 (R. leg), *Sinorhizobium fredii* HN01 (S. fre), *Mesorhizobium loti* 541 (M. lot), *Sinorhizobium arboris* HAMBI 1552(S. arb), *Azorhizobium caulinodans* USDA 4892 (A. cau). (A) the electrophoresis map of *Bam*HI-digested genomic DNA. (B) the electrophoresis map of *Pst*I-digested genomic DNA. (C) *Bam*HI-digested DNA hybridised with probe *dmtH*. (D) *Pst*I-digested DNA hybridised with probe dmtH. The Southern blot shows that *dmtH* gene is present in *R. leguminosarum* LRP 5045 and *M. loti* 541.
(TIF)

Figure S4 **HPLC of the pure standard MK-4, MK-7, MK-9 and UQ-10.**
(TIF)

Figure S5 **Mass spectrum of quinine compounds from** ***E.coli*** **JC7623Δ4-1 and the reacting system of testing DmtH peotein activity.** (A) Mass spectrum of DMK-8 which was accumulated in *E.coli* JC7623**Δ**4-1 but no MK-8′ MS found. (B) Mass spectrum of MK-8 which was tested from in reaction system. The result showed MK-8 was synthesized in the reaction system and DmtH had the the function of methyltransferase.
(TIF)
