# Supplementary figures and images for: Identification of a Novel Gene for Biosynthesis of a Bacteroid-Specific Electron Carrier Menaquinone
Source: PLoS One. 2011 Dec 14;6(12):e28995. doi: 10.1371/journal.pone.0028995 (PMC3237581; doi:10.1371/journal.pone.0028995)

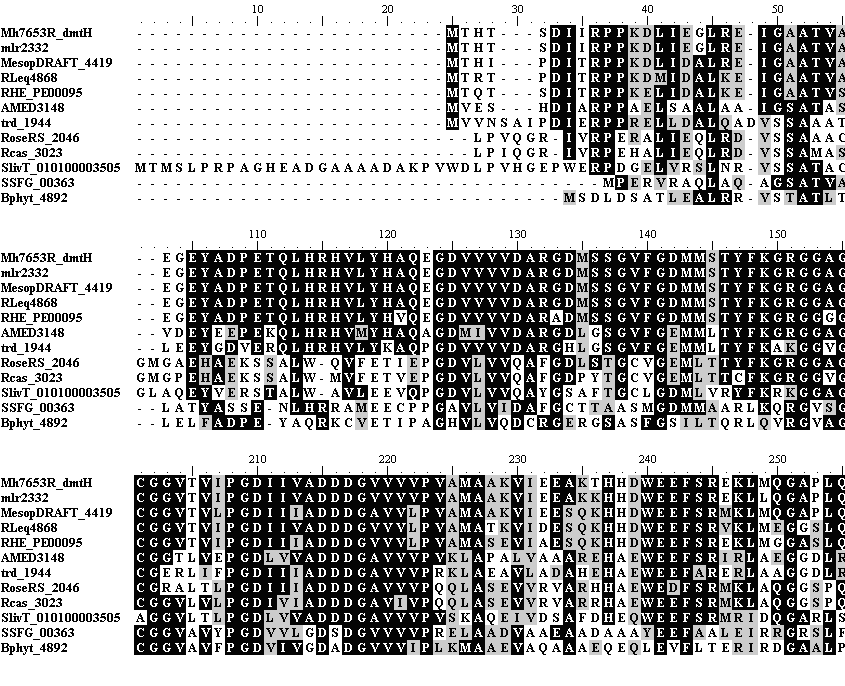

Supplement: Figure S1 — Multiple sequence alignment of the DmtH protein and the homologous sequence. Columns that are dark represent identical residues, lighter black represent similar residues in function. The numbers on the upper indicate the positions of amino acids. Protein names are indicated to the left of the alignment. These proteins come from Moserhizobium. loti MAFF303099 (mlr2332), Moserhizobium opportunistum WSM2075 (MesopDRAFT_4419), Rhizobium leguminosarum bv. viciae WSM1325 (RLeq4868), Rhizobium etli CFN42 (RHE_PE00095), Amycolatopsis mediterranei U32 (AMED3148), Roseiflexus sp. RS-1 (RoseRS_2046), Thermomicrobium roseum DSM 5159 (trd_1944), Roseiflexus castenholzii DSM 13941 (Rcas_3023), Streptomyces lividans TK24 (SlivT_010100003505), Streptomyces ghanaensis ATCC 14672 (SSFG_00363), Burkholderia phytofirmans PsJN (Bphyt_4892). (TIF) [file pone.0028995.s001.tif]

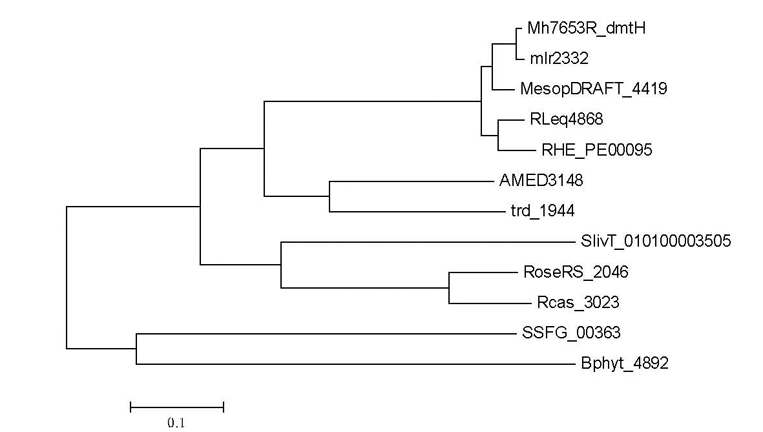

Supplement: Figure S2 — Homology tree of DmtH homologs. The homology tree was constructed using MEGA5.04 software (Tamura K, 2011). The scale bar indicates 10% substitutions per site. (TIF) [file pone.0028995.s002.tif]

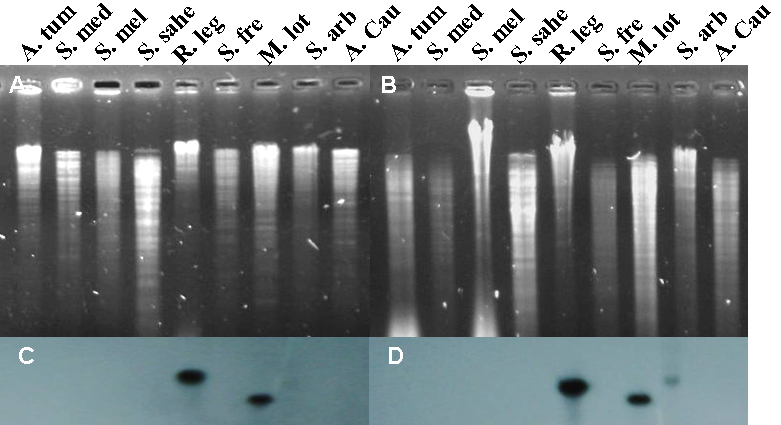

Supplement: Figure S3 — Southern blot analysis of dmtH gene in different Rhizobium genomes. The total genomic DNA of tested strains were digested for at least 3 h with 5-10 U of restriction enzyme PstI and BamHI per µg of DNA. Digested genomes were subjected to electrophoresis and transfer to nylon membranes, then hybridized to a 32P-labeled probe. The tested strains was Agrobacterium tumefaciense IAMI 3129 (A. tum), Sinorhizobium medicae USDA 1037 (S. med), Sinorhizobium meliloti USDA 2011 (S. mel), Sinorhizobium saheli LMG 7837 (S. sah), Rhizobium leguminosarum LRP 5045 (R. leg), Sinorhizobium fredii HN01 (S. fre), Mesorhizobium loti 541 (M. lot), Sinorhizobium arboris HAMBI 1552(S. arb), Azorhizobium caulinodans USDA 4892 (A. cau). (A) the electrophoresis map of BamHI-digested genomic DNA. (B) the electrophoresis map of PstI-digested genomic DNA. (C) BamHI-digested DNA hybridised with probe dmtH. (D) PstI-digested DNA hybridised with probe dmtH. The Southern blot shows that dmtH gene is present in R. leguminosarum LRP 5045 and M. loti 541. (TIF) [file pone.0028995.s003.tif]

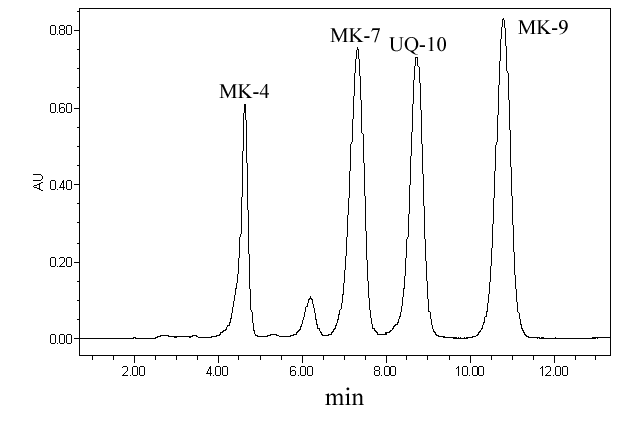

Supplement: Figure S4 — HPLC of the pure standard MK-4, MK-7, MK-9 and UQ-10. (TIF) [file pone.0028995.s004.tif]

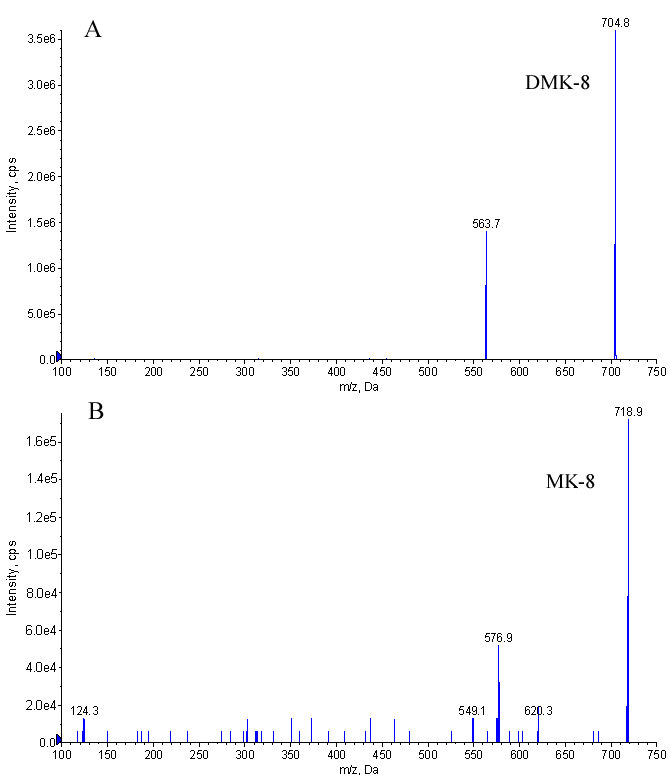

Supplement: Figure S5 — Mass spectrum of quinine compounds from E.coli JC7623Δ4-1 and the reacting system of testing DmtH peotein activity. (A) Mass spectrum of DMK-8 which was accumulated in E.coli JC7623Δ4-1 but no MK-8′ MS found. (B) Mass spectrum of MK-8 which was tested from in reaction system. The result showed MK-8 was synthesized in the reaction system and DmtH had the the function of methyltransferase. (TIF) [file pone.0028995.s005.tif]
